# Management of Pregnancy with Klippel-Trenaunay-Weber Syndrome: A Case Report and Review

**DOI:** 10.1155/2018/6583562

**Published:** 2018-07-12

**Authors:** Rati Chadha

**Affiliations:** Department of Obstetrics and Gynecology; Division of Maternal Fetal Medicine, Foothills Medical Center, University of Calgary, Calgary, AB, Canada

## Abstract

**Background:**

Klippel-Trenaunay-Weber syndrome is a rare neurocutaneous syndrome with vascular involvement. Given the rarity of the syndrome, its management in pregnancy is based on the outcome of a few case reports and expert opinion.

**Case Summary:**

The management of a complicated case with its antepartum, intrapartum, and postpartum concerns has been addressed in this review.

**Conclusions:**

Prenatal consults with anesthesia, general surgery, intervention radiology, and internal medicine should be arranged, prior to delivery in anticipation of all the possible complications. Apart from the pregnancy management, preconceptional counselling including the genetics, prognosis, and contraception has an important role in patient management.

## 1. Introduction

Klippel-Trenaunay Type Syndrome (KTTS) was first described in 1900 by Klippel and Trenaunay, who described it as a syndrome of osteohypertrophic varicose nevus [1]. In 1918, Weber added arteriovenous fistulae to the syndrome. It is therefore interchangeably used as KTS (Klippel-Trenaunay Syndrome) or KTW (Klippel-Trenaunay-Weber) syndrome.

KTW is a rare congenital abnormality with a variable expression [1] and an unknown etiology. The reported incidence is approximately at 1: 27500 newborns [2]. The triad that characterizes the syndrome consists of vascular skin nevus, varicose veins or venous malformations, and asymmetric hypertrophy of soft tissue and bone [2, 3]. Varicose veins or venous malformations are the most characteristic features. At least two of the three main symptoms should be present to accept the diagnosis [4]. Other features that may be seen are hyperhidrosis, scoliosis and gait abnormalities, and neuroaxial venous malformations [5].

KTS is a neurocutaneous syndrome with vascular involvement [3]. There are many theories regarding the etiology of this syndrome. It is most likely the result of a somatic mosaicism: a postzygotic mutation that only affects a subset of the cells within the body [2].

A large lateral varice is so common that it is commonly referred to the Klippel-Trenaunay vein or the Vein of Serville [6]. This vein invariably begins at the foot; tunnels laterally past the knee and then courses medially at the level of the groin. Internal iliac vein abnormalities can lead to varicosities around the colon, rectum, uterus, and bladder. The diagnosis of this disease is usually initiated by the clinical appearance in childhood [1]. During pregnancy the venous malformations increase with pelvic and intra-abdominal involvement [1]. The complications, therefore, seen are venous insufficiency, cellulitis, ulcers, thrombophlebitis, thromboembolism, lymphangiectasia [7], consumptive coagulopathy with severe thrombocytopenia (Kasabach-Merritt syndrome), and increased bleeding in the intrapartum period [8–11].

We have discussed a case of KTW syndrome below with multiple antepartum complications and their management.

## 2. Case Report

Our patient is a 19 yo G1P0 with an unplanned pregnancy, who was seen for the 1st time at 20+4 weeks, with syncope and right sided chest pain. Pulmonary embolism was confirmed on a spiral CT scan.

Prior to the current pregnancy, she had an extensive history of emergency room (ER) visits and pediatric intensive care unit admissions for recurrent cellulitis and septic shock secondary to lymphedema. She had been noncompliant with oral suppressive antibiotics. She had also developed toxic shock syndrome secondary to Group A streptococcal cellulitis and had multiple debridements with skin grafts as a result of necrotising fasciitis. Compounding her risk of infections was her picking behavior secondary to anxiety.

She had required ECMO (extracorporeal membrane oxygenation) for severe biventricular dysfunction from presumed septic cardiomyopathy; peritoneal hemodialysis for acute renal failure and multiple episodes of mechanical ventilation and aggressive vasopressor inotropic support.

Seven months prior to her pregnancy diagnosis, she had a bladder rupture and peritonitis after self-insertion of a pen into her urethra, requiring laparotomy. Numerous large venous bleeders were encountered in the subcutaneous tissue related to her known KTW syndrome.

She was also smoking about half to one pack of cigarettes and multiple joints of marijuana since the age of 13 years. Her overall social situation was tenuous with limited resources for food, housing, family support, and a borderline personality disorder.

**Figure 1:**
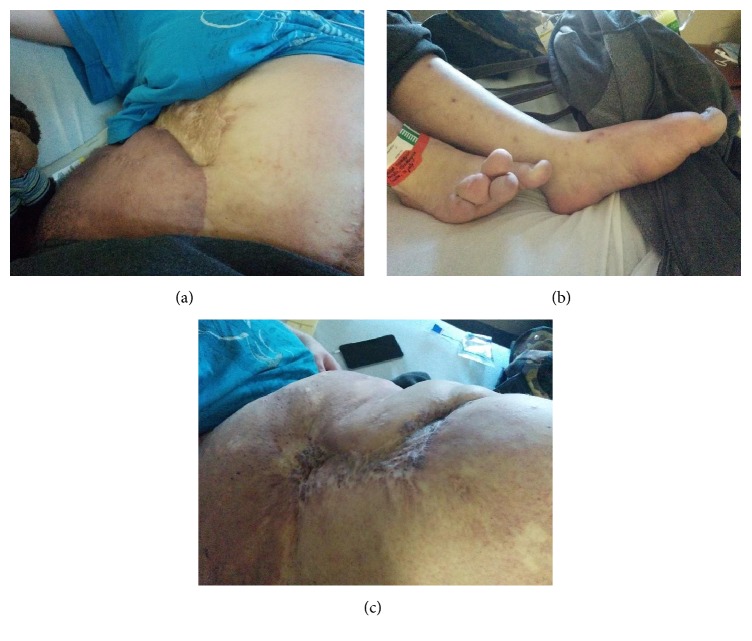
(a) Extremely large port wine stain/hemangioma extending from the right flank to midthigh with superficial excoriations. (b) Toes enlarged to about 5 cm in width and overlapping with each other. (c) Midline laparotomy scar along with multiple other scars from the debridements.

At the time of presentation, her resting heart rate was 110/min and increased to 160/min on standing. On examination, she had an extremely large port wine stain extending from the right flank to midthigh with superficial excoriations (Figure 1(a)). Bilateral significant lymphedema to the lower extremities was noted. The toes were enlarged to about 5 cm in width and were overlapping with each other (Figure 1(b)). Over her abdomen she had a midline laparotomy scar that had healed along with multiple other scars from the debridements (Figure 1(c)). Her gait was abnormal, secondary to the severe scoliosis.

She was started on therapeutic enoxaparin, with the dose being adjusted on the basis of the antifactor Xa levels (goal of 0.5-1.0). Her syncope was attributed to the significant lymphedema and venous varicosities in the lower extremities, along with the consumption of very high-glucose containing fluids (juice and pop) resulting in osmotic diuresis. She also had an ECHO done which demonstrated low left ventricular function. Her compliance as an outpatient for taking heparin and antibiotics was poor. The decision was made, therefore, to keep her as an inpatient until delivery from about 24 weeks, which greatly improved her nutrition and compliance, as all medications were given under nursing supervision.

She was seen by psychiatry and social work while in the hospital. She was counselled by prenatal genetics but declined testing for herself or on her newborn.

**Figure 2:**
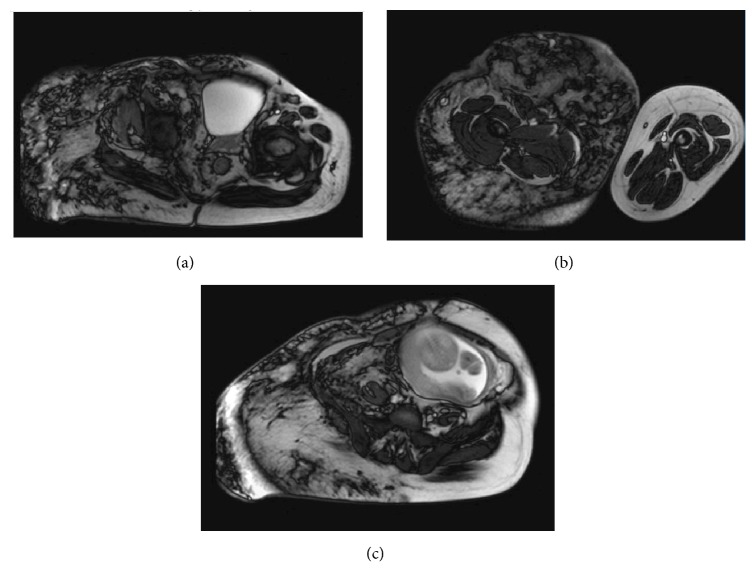
**MRI images depicting**. (a) Marked hemihypertrophy of the subcutaneous soft tissues on the right side at the level of the lower abdomen. (b) Marked hemihypertrophy of the subcutaneous soft tissues on the right thigh as compared to the left (normal). (c) Numerous venous channels were noted to cross at the potential epidural injection sites from L1 to L5. The thickness from the skin to the thecal space was 9.4 cm.

An MRI of the abdomen and pelvis performed revealed marked hemihypertrophy of the subcutaneous soft tissues on the right side at the level of the lower abdomen (Figure 2(a)); pelvis and buttock and thigh region (Figure 2(b)), which contain innumerable venous vascular malformations and varicosities. No increase in vascularity was noted in perineal or uterine area. These changes were consistent with KTW syndrome.

The MRI of the spine revealed marked levoconvex scoliosis with a marked leftward pelvic tilt. A developmentally narrow spinal canal was noted. Numerous venous channels were noted to cross at the potential epidural injection sites from L1 to L5. The thickness from the skin to the thecal space was 9.4 cm (Figure 2(c)). No intracranial or intraspinal vascular malformations were noted. Once the MRI was completed, anesthesiology was consulted. Their recommendation was for extreme caution for neuroaxial anesthesia and if to be given to be performed above the level of L1. Given the increased risk of disseminated intravascular coagulation (DIC), several units of blood were to be cross-matched with two large bore intravenous access. Consideration for the use of cell-saver and the availability of a Trauma Pak if bleeding was a concern was proposed.

Serial scans on the fetus demonstrated as decrease in growth and along with a persistent breech presentation. A decision was made for an elective caesarian section at 37+3 weeks, given the intrauterine growth restriction with abnormal Doppler in a breech presentation. A multidisciplinary discussion between general surgery, neonatology, anesthesia, internal medicine, and nursing was arranged.

Enoxaparin was discontinued 24 hours prior to the elected time of the C-section. Four units of packed red blood cells had been placed in the OR in the event that there was a need to transfuse the patient. General anesthesia under fiber optic visualization was administrated. A midline vertical incision was made. Extensive varicosities were then found in the subcutaneous tissues closer to the level of the pelvis, which were ligated. The incision was extended superiorly to be away from the varicosities. The uterus was found to be bicornuate, which explained the breech presentation of the fetus. The neonate was at the 10th %ile for growth. No obvious stigmata of KTW syndrome were noted at the time of birth.

The patient had been counselled regarding the placement of a MIRENA IUD in the uterus at the end of the C-section for a reliable form of contraception. However, she declined contraception. She was discharged on Day# 3 with transition on to warfarin for six months. Unfortunately, given the limited compliance, she had another pulmonary embolism four months after delivery.

## 3. Discussion

Given the paucity of literature on this unique condition, in this case report, we have attempted to discuss the antenatal, intrapartum and postpartum management under specific areas as mentioned below, with the aim of guiding management for other healthcare professionals when such a case presents.

### 3.1. Genetics

Most cases are sporadic in origin. On review of literature there are a handful of familial cases of KTW syndrome. A case of reciprocal translocation [46, XX, t(5;11)(q13.3;p15.10], suggesting that potential aetiology could be a single gene disorder [12]. However, Aelvoet [13] described a multifactorial genetic form. A somatic mutation of a factor required for vasculogenesis and angiogenesis in embryonic development has been thought to cause hypertrophy of the soft tissue and bone.

The other etiology is that there is overexpression of insulin-like growth factor-2 which causes tissue hypertrophy in different overgrowth disorders, including KTW syndrome. This was elucidated by Speradeo [14] when he described a family in which there was a case of KTW syndrome and a first cousin with Beckwith-Wiedemann syndrome.

For our patient there was no available family history and she declined genetic testing for herself.

### 3.2. Prenatal Diagnosis

There have been multiple case reports of prenatal diagnosis of this condition. The presentation may be in the form of multiple echo lucent areas suggestive of cutaneous vascular malformations [15] and/or hemihypertrophy of a lower extremity [16]. Roberts [17] described a case where by serial sonography was used to follow the in utero progression of the case and helped to decide the mode of delivery as a C-section, given the risk of labour dystocia and excessive fetal bleeding. In the second trimester the dominant abnormality was the cutaneous vascular lesions, while in the third trimester limb hypertrophy was the predominant finding. Other case reports have described findings of nonimmune hydrops [18] and cardiomegaly [19], secondary to the vascular malformations.

Differential diagnosis for this presentation on ultrasound is multiple congenital teratoma and cystic hygroma.

In addition, it has been suggested that fetuses in patients with KTW may be at increased risk of developing intrauterine growth restriction [20].

MRI has been extensively used in the management of KTW syndrome in children and adults. There is a case report of the prenatal use of MRI to confirm the diagnosis [21].

Our patient had multiple ultrasounds to assess fetal growth and to determine if there were any signs of a similar disorder in the fetus. No obvious cutaneous or bony anomalies were noted.

### 3.3. Prognosis

The main cause of complications is the presence of vascular anomalies which can cause cellulitis and resulting ulcers, sepsis, necrotising fasciitis requiring skin debridement, and vascular malformations in abdomen and pelvis. The main complications associated in pregnancy and postpartum period are thromboembolism and hemorrhage. The use of thromboembolic prophylaxis with low molecular weight heparin is generally recommended in the antepartum and the postpartum period [8]. Consideration should be given to the placement of a temporary inferior vena cava (IVC) filter to decrease the risk of pulmonary embolism.

In a cross-sectional study, KTS-related symptoms were aggravated during pregnancy in 43% of patients. Deep vein thrombosis was present in 5.8% and pulmonary embolism was present in 2.3% of pregnancies, which was extremely high compared with the reference population (P < 0.0001) [11].

Preterm labour has also been reported more often in patients with KTS [9]. However, in a cross-sectional study [11], this was not found.

Multiple complications such as pulmonary embolism, cellulitis, and necrotising fasciitis had occurred in our patient. However, the antepartum course after presentation to the hospital was uneventful.

### 3.4. Anesthesia

A MRI of the spine is recommended in the 3^rd^ trimester to determine if neuroaxial anesthesia can be administered. In many cases such as in ours, due to the associated scoliosis or if there is a concern that a vascular malformation in the peridural space that may have been overlooked, general anesthesia is resorted to. However, general anesthesia is not without concern. In case of difficult airway secondary to vascular malformations, consideration for the use of awake fiber-optic intubation techniques to secure the airway [22] or the use of a laryngeal mask airway with continuous cricoid pressure, in an effort to minimize airway trauma and hemorrhage is recommended [23].

Additionally, given the risk of hemorrhage, arrangements for packed red blood cell and clotting factors transfusion or cell-saver technology should be made. The coagulation status of the patient should also be checked prior to delivery along with serial complete blood counts.

Getting an anesthesia consult helped the team be prepared for any complications. Fortunately, in our case no hemorrhage or another anesthetic or surgical complication was noted.

### 3.5. Mode of Delivery

The mode of delivery needs to be individualized per case. This will depend upon the imaging findings for the presence of vascular abnormalities in the pelvis and abdomen. The presence of varicosities/vascular malformations in the cervical, vaginal, or vulvar area will make vaginal delivery difficult because of the risk of rupture and hemorrhage. The vascular malformations are also associated with an increased risk of cerebrovascular accidents [24] in patients with intracranial anomalies. In such cases, operative vaginal delivery is recommended. If a caesarian section is the decided mode of delivery, intra-abdominal varicosities may require a midline vertical incision, such as in our patient. Disruption of the superficial draining veins of a leg during a Caesarean delivery may result in eventual amputation due to unrelieved venous engorgement [22].

### 3.6. Birth Control

Given the risk of thromboembolism even in the nonpregnant state, estrogen based contraception should be avoided. Depo-Provera or intrauterine device (progesterone or the copper) is good options.

Our patient received extensive counselling for contraception; however she declined to use any reliable method.

### 3.7. Preconceptional Counselling

Even though a few cases of familial occurrences have been described, the risk of transmission from an affected mother to her infant is unknown. There is evidence of an increased prevalence of vascular malformations in family members of these patients. Adequate counselling regarding antepartum and intrapartum complications as described above is recommended.

## 4. Conclusions

Less than hundred cases of pregnancies with KTW syndrome have been reported [11]. Pregnancy has historically been discouraged in women with KTW syndrome given the increased risk of complications. Prenatal consults with anesthesia, general surgery, intervention radiology, and internal medicine should be arranged prior to delivery in anticipation of all the possible complications. Therefore, an individualized multidisciplinary plan will help mitigate the complications associated with this condition for optimal results.

## Abbreviations

*KTTS*: * Klippel-Trenaunay Type Syndrome*
*KTS*: * Klippel-Trenaunay Syndrome*
*KTW*: * Klippel-Trenaunay-Weber*
*G1P0*: * Gravida 1 para 0*
*CT*: *Computerized tomography*
*ECMO*: * Extracorporeal membrane oxygenation*
*ECHO*: * Echocardiography*
*MRI*: * Magnetic resonance imaging*
*DIC*: * Disseminated intravascular coagulation*
*IUD*: * Intrauterine device*
*IVC*: * Inferior vena cava*.
